# Sodium Decanoate Improves Intestinal Epithelial Barrier and Antioxidation via Activating G Protein-Coupled Receptor-43

**DOI:** 10.3390/nu13082756

**Published:** 2021-08-11

**Authors:** Jinbiao Zhao, Jinhua Hu, Xi Ma

**Affiliations:** State Key Laboratory of Animal Nutrition, College of Animal Science and Technology, China Agricultural University, Beijing 100193, China; jinbiaozhao@cau.edu.cn (J.Z.); 15600911358@163.com (J.H.)

**Keywords:** antioxidant, intestinal barrier, microbiota, decanoic acid, gut health

## Abstract

The study was conducted to explore actions of decanoic acid on regulating intestinal barrier and antioxidant functions in intestinal epithelium cells isolated from porcine jejunum (IPEC-J2) and C57/BL6 mice models. In vitro and vivo assays, mice and IPEC-J2 cells treated by H_2_O_2_ were disposed of sodium decanoate and sodium butyrate to determine intestinal barrier and antioxidant functions of the host. Results showed that sodium decanoate upregulated expression of tight junction proteins and improved antioxidant capacity in both IPEC-J2 cells treated by H_2_O_2_ and mice models (*p* < 0.05). Sodium decanoate increased weight gain and ileal villus height of mice compared with control and sodium butyrate treatments (*p* < 0.05). Sodium decanoate increased α-diversity of ileal microbiota, volatile fatty acids concentration, and G protein-coupled receptor-43 (GPR-43) expression in the ileum and colon of mice (*p* < 0.05). In conclusion, sodium decanoate improved antioxidant capacity, intestinal morphology, and gut physical barrier of intestinal epithelial cells, resulting in an increase growth performance of mice, which is mediated through activating GPR-43 signaling.

## 1. Introduction

Medium chain fatty acids (MCFAs) are saturated molecules containing 6~12 carbons, mainly including hexanoic acid, caprylic acid, decanoic acid, and lauric acid. Even-numbered MCFAs are naturally present in food, including coconut oil, palm kernel, cow’s milk, and human breast milk [1,2]. In contrast, odd-numbered MCFAs are primarily chemically-synthesized from the available precursors [3]. MCFAs have proven to increase growth performance, improve intestinal immunological functions, and exhibit a broad-spectrum antimicrobial activity against a range of bacterial species, resulting in an improvement of gut health [4]. Diets supplemented with 0.3% caprylic acid or decanoic acid improved daily weight gain of piglets in d 0 to 28 of post-wean [5]. An improvement of growth performance in weanling piglets could be associated with changes of gut microbiota shaped by caprylic acid or decanoic acid [6]. Previous studies used the intestinal epithelium cells isolated from porcine jejunum (IPEC-J2) challenged with *Escherichia coli* ATCC 43889 and reported that the treatment with caprylic acid significantly reduced bacterial translocation, enhanced antibacterial activity, and remarkably increased secretion of porcine β-defensins 1 (pBD-1) and pBD-2 [3]. However, in this publication, caprylic acid supplementation had no positive responses on the intestinal barrier function of IPEC-J2, such as mRNA and protein expression of ZO-1 and occludin. In addition, a treatment of caprylic acid suppressed a secretion of interleukin-8 from Caco-2 cells in vitro, but decanoic acid increased production of interleukin-8 secreted by Caco-2 [7], which indicated that there are some differences in biological functions on intestinal health and immunological functions among diverse varieties of MCFAs. 

In practice, antibiotics are always supplemented into infant and young animals to prevent and cure clinical symptoms of gastrointestinal diseases at weaning due to the oxidation stress. Unfortunately, abuse of antibiotics in weanling pig nutrition has led to some severe problems, such as emergences of drug-resistant genes and “super” bacteria, food safety, and environmental pollution [8]. At present, antibiotics have been banned to supplement in livestock production in China, Europe, and America. Young animals and infants at weaning would suffer a series of severe oxidation stress from changes of environment and dietary nutrition, leading to injury of intestinal barrier and disorder of gut microbial community, and then result in lower performance, clinical diarrhea, and even death [9,10]. Therefore, it is vital and urgent to exploit some products as antibiotic alternatives to improve gut health and growth performance of piglets suffering from weaning stress. Many studies have reported that butyric acid can be absorbed by intestinal epithelial cells to improve intestinal barrier function and antioxidant capacity by mediating G-protein couple receptors (GPR) [11,12]. Recently, our cooperative institution reported that dietary supplementation with MCFA as the replacement of zinc oxide improved the immune function of the intestine and gut microbiota community in weanling pigs’ model, resulting in the improved growth performance [13]. However, potential mechanisms of MCFA on regulating intestinal health of the weanling pigs have been unclear. We hypothesized that decanoic acid improves intestinal barrier function and antioxidant capacity of the host by activating GPRS to relieve damage of the oxidation stress. Based on the previous finding above, the objective of this study was to continuously exploit the effects of decanoic acid on intestinal barrier and antioxidant functions in IPEC-J2 of pig and C57/BL6 mice models in comparison with butyrate acid, in order to look for a potential anbiotic alternative in human and animal nutrition.

## 2. Materials and Methods

### 2.1. Experimental Design

All of the experimental protocols were carried out with approval of the China Agricultural University Animal Care and Use Committee (CAU20201247-1). In vitro, the effect of decanoic acid was studied on tight junction proteins expression and antioxidant functions of IPEC-J2. Additionally, 0.1, 0.5, 1, 2, and 5 mmol/L of sodium decanoate and sodium butyrate were used to select an optimal treatment concentration according to the cell viability of IPEC-J2, and finally 1 mmol/L of sodium decanoate and 0.5 mmol/L of sodium butyrate were implemented to the treatment of normal IPEC-J2 for 24 h to determine expression of tight junction proteins. Meanwhile, IPEC-J2 cells were treated by different concentrations of 0.1, 0.3, 0.5, 0.7, 0.9, 1.1, and 1.3 μmol/L H_2_O_2_ and different treatment periods of 1, 2, and 4 h were used to select an optimal treatment concentration and time points. At last, IPEC-J2 cells were treated with 0.7 μmol/L H_2_O_2_ for 2 h to establish the oxidative damage model of the cells, and damaged IPEC-J2 cells were further treated by 1 mmol/L of sodium decanoate and 0.5 mmol/L of sodium butyrate for 24 h to determine antioxidant functions. 

In vivo trial, 36 C57/BL6 mice with weaning age of 21 d were allocated into three dietary treatments randomly, which are control (CON), 5 g/kg sodium decanoate, and 5 g/kg sodium butyrate diets, respectively, to evaluate the effects of sodium decanoate on growth performance, antioxidant capacity, intestinal barrier, and gut microbiota in mice. Each dietary treatment included 6 cages (replicates), and 2 mice per replicate. This feeding trial of mice lasted 28 d, and the feed intake and body weight of mice were measured on d 28 to calculate the average daily feed intake (ADFI), average daily gain (ADG), and ratio of feed to gain. In addition, one mouse from each cage was euthanized to collect blood samples from the eyes and intestinal tissue and mucosal samples to evaluate functions of antioxidant capacity, gut morphology, and intestinal barriers. Meanwhile, ileal and colonic digesta of mice were also collected to analyze the VFA concentration and microbial community. The diets formulation of mice is presented in Supplementary Table S1.

### 2.2. Samples Collection

The IPEC-J2 cells were cultured in a medium formulated with 85% DMEM-F12 and 15% FBS. When the cells have fused to about 50%, the IPEC-J2 cells were treated with sodium decanoic and sodium butyrate for 24 h. Additionally, IPEC-J2 cells were treated with H_2_O_2_ for 1, 2, 4 h under the same culture medium condition. After removing the culture solution, the cells were rinsed by the medium. A plate was added with six holes of 200 μL 1.5× protein sample buffer to pyrolysis cells, and then transferred to a 1.5 mL centrifuge tube. The cells were heated by the boiling water bath for 10 min and rapid cooled for 30 s on ice. Finally, the treated samples were stored at −20 °C for further analysis of tight junction proteins expression. In addition, IPEC-J2 cells obtained after the culture were treated with a 0.5 mL PBS solution to make cell suspension. Then, they were crushed on ice for 8 min using an ultrasonic cracker to extract the cell protein. Cellular proteins were measured at 562 nm using the BCA protein assay kit according to the manufacturer’s instructions. The extracted protein samples were stored at −20 °C for analysis of the antioxidant enzymes activity.

Each intestinal sample was collected from the approximately middle positions in the ileum and colon of mice, respectively. The obtained tissues of ileum and colon were rinsed with PBS to remove the intestinal contents and fixed in 4% paraformaldehyde for the analysis of H&E staining. In addition, a portion of intestinal mucosa was frozen in liquid nitrogen immediately and then stored at −80 °C before microbial genomic DNA extraction. Blood samples were collected in 10 mL anti-coagulant tubes and centrifuged at 3500 rpm at 4 °C for 15 min for the collection of plasma. The plasma was stored at −80 °C for further analysis. 

### 2.3. Chemical and Reagents

The IPEC-J2 cells were kindly provided by the ministry of Agriculture and Rural Affairs Feed Industry Centre (Beijing, China). Sodium decanoate, sodium butyrate, and H_2_O_2_ were brought from Sigma-Aldrich (St. Louis, MI, USA). The IPEC-J2 medium of DMEM-F12 and fetal bovine serum (FBS) were purchased from Gibco (Grand Island, NY, USA). The cell vitality detection kit (cell counting kit-8, CCK 8) was purchased from Solarbio (Beijing, China) and antioxidation detection kits were purchased from Nanjing Jiancheng Bioengineering Institute (Nanjing, China). The protein marker of Western blot was purchased from Mei5 Biotechnology (Beijing, China), the detection of intestinal barrier proteins were brought from Abcam (Shanghai, China), and TM800 infrared labelled anti-rabbit secondary antibodies and anti-mouse from goats were purchased from LI-COR (Lincoln, NE, USA).

### 2.4. Laboratory Analysis

#### 2.4.1. Cell Viability

The IPEC-J2 cells were cultured in a medium formulated with 85% DMEM/F-12 and 15% FBS, and treated by saline, sodium decanoate, sodium butyrate, and H_2_O_2_ when the cells have fused to about 60%. Cell viability was monitored using a 3-(4,5-dimethylthiazol-2-yl)-2,5-diphenyltetrazolium bromide (MTT) colorimetric assay. 

The cells were seeded into 96-well plates with 1 × 10^5^ per well in a 100 μL culture medium and incubated for 24 h. Then, they were exposed to different concentrations of sodium decanoate and sodium butyrate at 0, 0.1, 0.5, 1, 2, and 5 mmol/L for 24 h and 10 μL of 5 mg/mL MTT added prior to culturing for 4 h. Meanwhile, the IPEC-J2 cells were treated with different levels of H_2_O_2_ at 0, 0.1, 0.3, 0.5, 0.7, 0.9, 1.1, and 1.3 μmol/L for 1, 2, and 4 h to build an oxidant damage model. Then, the 96-well plates were irradiated with a laser (808 nm, 0.5 W cm^−2^) for 8 min, after which the cells were still incubated for another 12 h. Subsequently, 10 μL of the MTT solution (5 mg mL^−1^ in PBS) was added and incubated for 4 h. The supernatant was removed and the culture resuspended in 150 μL of isopropanol to dissolve the MTT formazan. Absorbance was recorded at 490 nm with a spectrophotometer (Biomate 5, Thermo Electron Corporation, Rochester, NY, USA). Optimal treatments of sodium decanoate, sodium butyrate, and H_2_O_2_ were chosen and used in the following experiments. The effects of sodium decanoate and sodium butyrate on the viability of IPEC-J2 treated by H_2_O_2_ were assessed by comparing the percentage of viable cells to that of vehicle-treated control cells. The control cells were arbitrarily set as 100% viability. The cell viability was calculated by the following equation: Cell viability (%) = (A_treatment_/A_control_) × 100%.

#### 2.4.2. Intestinal Morphology

Haematoxylin and Eosin (H&E) staining was performed to analyze intestinal morphology using the previously described protocol [14]. Briefly, tissues of ileum and colon fixed with 4% formaldehyde were embedded in paraffin, and sections 5 μm in thickness were obtained and stained with hematoxylin and eosin. Digital images of intestinal morphology at 100× magnification were obtained using a light microscope. Finally, the villus height and crypt depth of the intestine in each image was measured.

#### 2.4.3. Expression of Tight Junction Proteins and GPR-43

The protein expression of claudin-1, claudin-3, claudin-7, occluding, and GPR-43 was measured using the Western blot (WB) analysis. Simply, the protein concentration in the IPEC-J2 cells extracts was determined using a BCA Protein Assay Kit. Extracts containing equal amounts of protein (30 μg) were resolved on 10% polyacrylamide gels and transferred onto PVDF membranes (Millipore, Billerica, MA, USA). The membrane was blocked for 3 h with 5% skimmed milk powder and incubated overnight with antibodies for claudin-1, claudin-3, claudin-7, occluding, GPR-43, and anti-β-actin in a 1:2000 dilution. After three washes, the secondary antibody was added in a 1:10,000 dilution and incubated at room temperature for 1 h. The membrane was washed three times and developed using WesternBright™ Peroxide (Advansta, San Jose, CA, USA) in an imaging system (Carestream, New York, NY, USA). The protein concentration was normalized to the amount of β-actin as an internal control.

#### 2.4.4. Antioxidant Indexes and Cytokines in Serum

Interleukin-1β (IL-1β), interleukin-6 (IL-2), tumor necrosis factor-α (TNF-α), malondialdehyde (MDA), superoxide dismutase (SOD), glutathione peroxidase (GPX), and catalase (CAT) activity were determined using enzyme-linked immunosorbent assay kits, according to the manufacturer’s instructions.

#### 2.4.5. Volatile Fatty Acids Concentration

Ileal and colonic digesta samples (0.5 g) were weighed into a 10 mL polypropylene tube and 8 mL of deionized water was added. After using an ultrasonic bath for 30 min, the mixture was centrifuged at 8000 rpm for 10 min. The suspension was diluted (1:50) with water and filtered through a 0.22 μm filter. A 25 µL sample solution was extracted and analyzed for the VFA, including acetic acid, propionic acid, and butyric acid by a HPLC (ICS-3000 Dionex, Sunnyvale, CA, USA), as described by Liu et al. [15]. 

#### 2.4.6. Microbial Community

Microbial DNA was extracted from ileal and colonic digesta samples using a DNA Kit (Omega Bio-tek, Norcross, GA, USA). A V4-V5 region of microbial 16S ribosomal RNA genes were amplified by PCR using primers 515F 5′-barcode- GTGCCAGCMGCCGCGG)-3′ and 907R 5′-CCGTCAATTCMTTTRAGTTT-3′. PCR reactions, amplicons purification and quantification, and raw data analysis were performed according to a previous literature [15]. Operational taxonomic units (OTUs) were clustered with 97% similarity cutoff using UPARSE and chimeric sequences were identified and removed using UCHIME. The taxonomy of each 16S rRNA gene sequence was analyzed by the RDP Classifier (http://rdp.cme.msu.edu/; Accessed date: 23 July 2018) against the silva (SSU128) 16S rRNA database using the confidence threshold of 70%. 

### 2.5. Statistical Analysis

All data were expressed as the mean ± standard deviation. The UNIVARIATE procedure of SAS 9.2 was used to check the normality of residuals and equal variances. Outliers were identified as any value that deviated from the treatment mean by ± 3 times of standard deviation. The statistical data analysis was carried out using the SAS 9.2 software (SAS Institute, Cary, NC, USA). Differences between groups were determined with the one-way analysis of variance (ANOVA), followed by Duncan’s multiple range test. Microbiota diversity metrics were performed from normalized OTU reads using R software (version 3.2.2) (Lucent Technologies, Murray Hill, NJ, USA). The significance level was set at *p* < 0.05, whereas 0.05 ≤ *p* < 0.10 was considered as a tendency.

## 3. Results

### 3.1. Optimal Treatment Concentrations of Sodium Decanoate and Its Effects on Tight Junction Proteins of IPEC-J2

**Figure 1:**
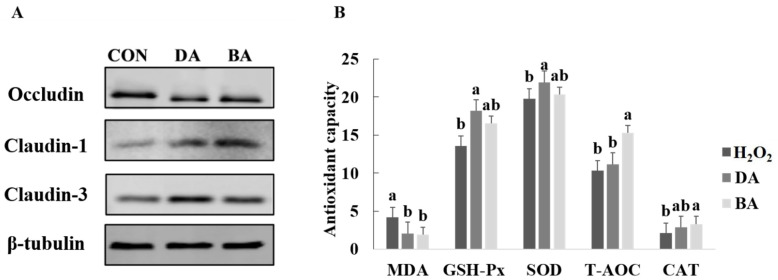
Effects of sodium decanoate and sodium butyrate on tight junction protein expression and antioxidant capacity in IPEC-J2 cells (Exp. 1). (**A**) Expression of tight junction proteins. (**B**) Antioxidase activity. A treatment concentration of 0.7 μmol/L H_2_O_2_ and a time treatment of 2 h were chosen to treat IPEC-J2 cells to develop a model of IPEC-J2 cells treated by H_2_O_2_. Concentration treatments of 1 mmol/L sodium decanoate and 0.5 mmol/L sodium butyrate were chosen to treat IPEC-J2 cells treated by H_2_O_2_. ^a,b^ Significant differences are indicated by different lowercase letters (*p* < 0.05). CON: The control diet; BA: The basal diet supplemented with 0.5% sodium butyrate; DA: The basal diet supplemented with 0.5% sodium decanoate.

Six concentration treatments of 0, 0.1, 0.5, 1, 2, and 5 mmol/L sodium decanoate or sodium butyrate were set in the experiment to select an appropriate concentration treatment on IPEC-J2 (Supplementary Figure S1). Cell viability was greatest at the treatment of 1 mmol/L sodium decanoate (*p* < 0.05). I n addition, cell viability was not significantly affected by a concentration of 0.5 mmol/L sodium butyrate, but it showed a dose-dependent decreasing trend in the concentration of 1, 2, and 5 mmol/L sodium butyrate. Therefore, concentration treatments of 1 mmol/L sodium decanoate and 0.5 mmol/L sodium butyrate were chosen to treat IPEC-J2. Results for protein expression of tight junction proteins after sodium decanoate and sodium butyrate treatments were shown in Figure 1A. There was no difference in protein expression of occludin from IPEC-J2 among CON, sodium decanoate, and sodium butyrate treatments. However, protein expression of claudin-3 and claudin-7 induced by sodium decanoate and sodium butyrate was shown as significantly greater than the CON group (*p* < 0.05).

### 3.2. Effects of Sodium Decanoate on Antioxidant Capacity and Tight Junction Protein Expression in an H_2_O_2_ Oxidant Damage Model of IPEC-J2

At first, six concentration treatments of 0.1, 0.3, 0.5, 0.7, 0.9, 1.1, and 1.3 μmol/L H_2_O_2_ and three times points of 1, 2, and 4 h were set in the experiment to select the appropriate concentration and time treatments on IPEC-J2 (Supplementary Figure S2). When the concentration of H_2_O_2_ was 0.7 μmol/L and the treatment time was 2 h, the cell viability of IPEC-J2 was significantly inhibited (*p* < 0.05). Therefore, a treatment concentration of 0.7 μmol/L H_2_O_2_ and a time treatment of 2 h were chosen to treat IPEC-J2. After the H_2_O_2_ treatment on IPEC-J2, 1 mmol/L sodium decanoate or 0.5 mmol/L sodium butyrate was provided to culture damaged cells, in order to study the cell morphology (Supplementary Figure S3). Results for antioxidant indexes of IPEC-J2 after sodium decanoate and sodium butyrate treatments are presented in Figure 1B. There were no differences in SOD and T-AOC among H_2_O_2_, sodium decanoate, and sodium butyrate treatments. However, sodium decanoate and sodium butyrate groups showed greater GSH-Px and CAT concentrations and lower MDA compared with the H_2_O_2_ treatment (*p* < 0.05).

### 3.3. Effects of Sodium Decanoate on Growth Performance, Intestinal Morphology, and Intestinal Barrier Functions in a Mice Model

**Figure 2:**
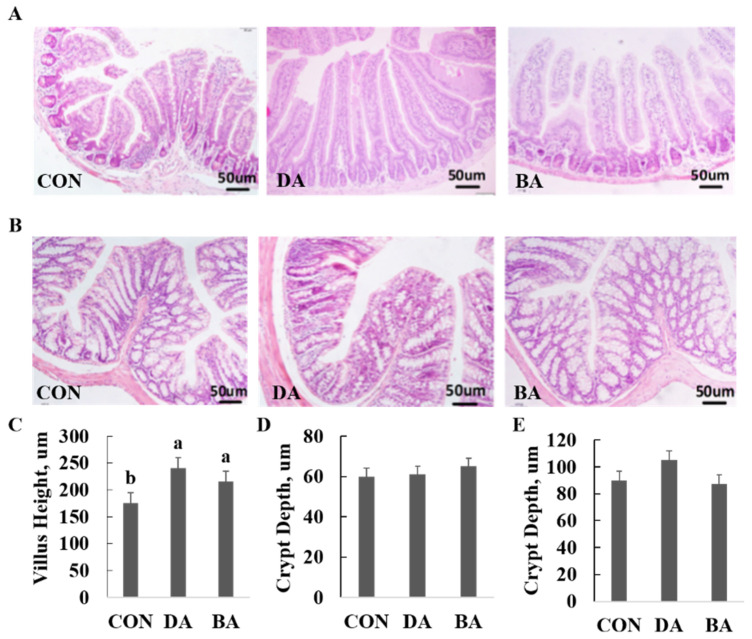
Effects of sodium decanoate and sodium butyrate on intestinal morphology in the ileum and colon of C57BL/6 mice (Exp. 2). (**A**) The representive image (*n* = 6). Intestinal morphology of the ileum in mice using H&E staining. (**B**) The representive image (*n* = 6). Intestinal morphology of the colon in mice using H&E staining. (**C**) Villus height of the ileum. (**D**) Crypt depth of the ileum. (**E**) Crypt depth of colon. A total of 36 mice with weaning age of 28 d were allocated into three dietary treatments randomly, which are control (CON), 5 g/kg sodium decanoate (DA), and 5 g/kg sodium butyrate (BA) diets. At the end, the intestinal tissue sample from each mouse was collected from the approximately middle positions in the ileum and colon to evaluate intestinal morphology. ^a,b^ Significant differences are indicated by different lowercase letters (*p* < 0.05).

A diet supplemented with 5 g/kg sodium decanoate or 5 g/kg sodium butyrate increased ADG and F:G ratio of mice compared with a CON diet (*p* < 0.05), but did not affect ADFI in a 28-d period after weaning (Table 1). Both 5 g/kg sodium decanoate and 5 g/kg sodium butyrate increased villus height in the ileum (*p* < 0.05), but did not affect crypt depth in the colon of mice (Figure 2).

### 3.4. Effects of Sodium Decanoate on Serum Antioxidant and Immunological Indexes in a Mice Model

**Figure 3:**
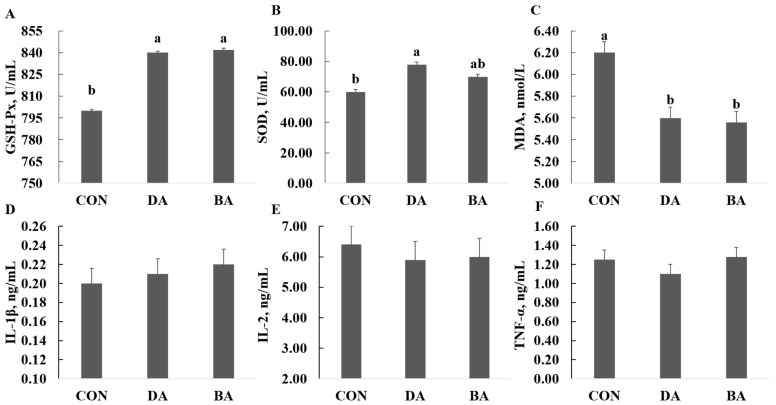
Effects of sodium decanoate and sodium butyrate on serum antioxidant and immune parameters in C57BL/6 mice (Exp. 2). (**A**) Serum GSH-Px. (**B**) Serum SOD. (**C**) Serum MDA. (**D**) Serum IL-1β. (**E**) Serum IL-2. (**F**) Serum TNF-α. A total of 36 mice with weaning age of 28 d were allocated into three dietary treatments randomly, which are control (CON), 5 g/kg sodium decanoate (DA), and 5 g/kg sodium butyrate (BA) diets. At the end, the blood sample from each mouse was collected to evaluate antioxidase activity and inflammatory cytokines levels. ^a,b^ Significant differences are indicated by different lowercase letters (*p* < 0.05), *n* = 6.

A diet supplemented with 5 g/kg sodium decanoate increased the concentrations of SOD and GSH-Px, but reduced the MDA content in the serum of mice (*p* < 0.05; Figure 3). Additionally, the treatment of sodium butyrate significantly increased the SOD concentration and decreased the MDA content in serum (*p* < 0.05). However, both 5 g/kg of sodium decanoate and 5 g/kg sodium butyrate had no influences on IL-β, TNF-α, and IL-2 in the serum of mice compared with the CON treatment.

### 3.5. Effects of Sodium Decanoate on Expression of Tight Junction Proteins and GPR-43 in the Intestine of Mice

**Figure 4:**
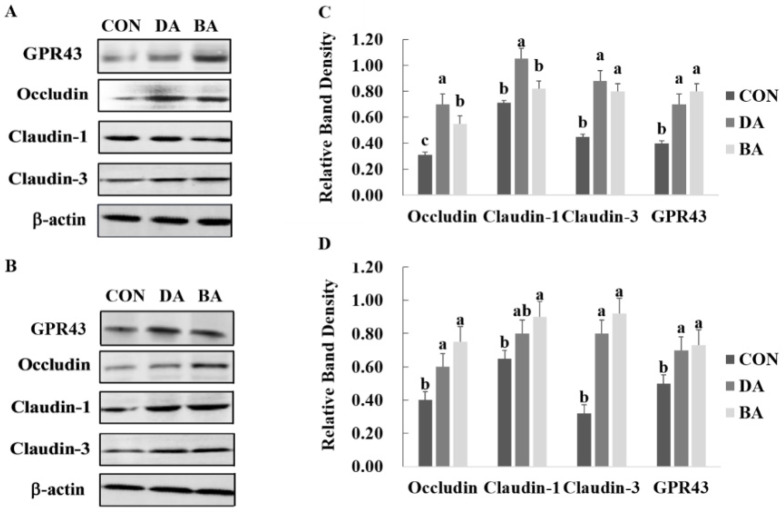
Effects of sodium decanoate and sodium butyrate on tight junction proteins and GPR-43 expressions in the ileal and colonic mucosa of C57BL/6 mice (Exp.2). (**A**) Expression of tight junction proteins and GPR-43 in the ileum. (**B**) Expression of tight junction proteins and GPR-43 in the colon. (**C**) Relative band density of tight junction proteins and GPR-43 in the ileum. (**D**) Relative band density of tight junction proteins and GPR-43 in the colon. A total of 36 mice with weaning age of 28 d were allocated into three dietary treatments randomly, which are control (CON), 5 g/kg sodium decanoate (DA), and 5 g/kg sodium butyrate (BA) diets. At the end, the intestinal mucosa sample from each mouse was collected from the approximately middle positions in the ileum and colon to evaluate the expression of tight junction proteins and G protein-coupled receptor-43. ^a,b^ Significant differences are indicated by different lowercase letters (*p* < 0.05).

The treatment of sodium decanoate improved the protein expression of claudin-1 and claudin-3 in the ileum, and occludin and claudin-3 in the colon (*p* < 0.05; Figure 4A). Additionally, the treatment of sodium butyrate improved the protein expression of occludin and claudin-3 in the ileum, and occludin, claudin-1, and claudin-3 in the colon of mice (*p* < 0.05). Furthermore, both groups of sodium decanoate and sodium butyrate improved the protein expression of GPR-43 in the ileum and colon of mice compared with the CON treatment (Figure 4B).

### 3.6. Effects of Sodium Decanoate on Gut Microbiota and Their Metabolites in a Mice Model

**Figure 5:**
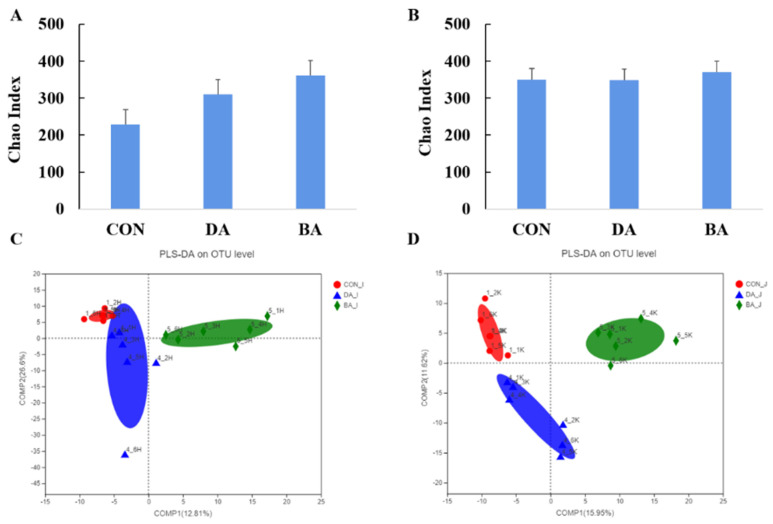
Effects of sodium decanoate and sodium butyrate on α and β diversity of intestinal flora of C57BL/6 mice (Exp. 2). (**A**) Chao index of microbiota in the ileum. (**B**) Chao index of microbiota in the colon. (**C**) PLS-DA analysis to β diversity in the ileum at the OTU level. (**D**) PLS-DA analysis to β diversity in the colon at the OTU level. A total of 36 mice with weaning age of 28 d were allocated into three dietary treatments randomly, which are control (CON), 5 g/kg sodium decanoate (DA), and 5 g/kg sodium butyrate (BA) diets. At the end, the intestinal digesta sample from each mouse was collected from the approximately middle positions in the ileum and colon to evaluate microbial community composition using high-throughput sequencing.

**Figure 6:**
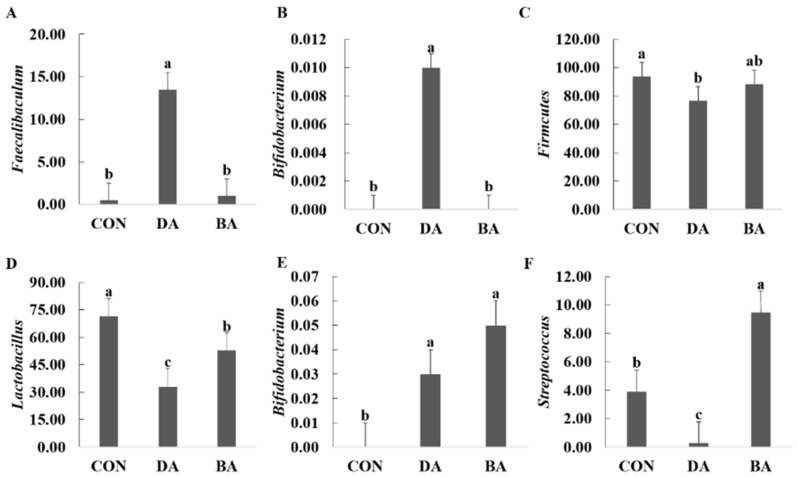
Effects of sodium decanoate and sodium butyrate on microflora structure in the ileum and colon of C57BL/6 mice at phylum and genus levels (Exp. 2). (**A**) An abundance of *Faecalibaculum* in the colon, %. (**B**) An abundance of *Bifidobacterium* in the colon, %. (**C**) An abundance of Firmcutes in the ileum, %. (**D**) An abundance of *Lactobacillus* in the ileum, %. (**E**) An abundance of *Bifidobacterium* in the ileum, %. (**F**) An abundance of *Streptococcus* in the ileum, %. A total of 36 mice with weaning age of 28 d were allocated into three dietary treatments randomly, which are control (CON), 5 g/kg sodium decanoate (DA), and 5 g/kg sodium butyrate (BA) diets. At the end, the intestinal digesta sample from each mouse was collected from the approximately middle positions in the ileum and colon to evaluate microbial community composition. Differential bacteria were analyzed using the R language. ^a,b,c^ Significant differences are indicated by different lowercase letters (*p* < 0.05), *n* = 6.

Both treatments of sodium decanoate and sodium butyrate increased the α-diversity of microbial community in the ileal and colonic digesta of mice (*p* < 0.05; Figure 5). Based on a result of microbial β-diversity, clusters of microbial composition in the ileal and colonic digesta can be distinguished significantly (*p* < 0.05). In addition, the differential microbial community shaped by 5 g/kg sodium decanoate and 5 g/kg sodium butyrate compared with the CON group were analyzed (Supplementary Figure S4). A diet supplementation with 5 g/kg sodium decanoate increased a population of Bacteroidates and decreased an abundance of Firmicutes (*p* < 0.05; Figure 6). To clarify further microbial compositions, effects of sodium decanoate on microbial community at a genus level were determined, and we found that 5 g/kg sodium decanoate decreased abundances of *Streptococcus* and *Lactobacillus*, but increased a population of *Bifidobacterium* in the ileum (*p* < 0.05). Importantly, the treatment of sodium decanoate increased abundances of *Faecalibaculum* and *Bifidobacterium* in the colon (*p* < 0.05).

Both treatments of sodium decanoate and sodium butyrate increased acetic acid, propionic acid, and butyric acid concentration in the ileal and colonic digesta of mice compared with the CON treatment (*p* < 0.05; Table 2).

## 4. Discussion

In the present study, diets supplemented with 5 g/kg sodium decanoate and 5 g/kg sodium butyrate improved the growth performance of mice. Those results are consistent with many previous reports that moderate inclusion levels of MCFAs could increase the growth performance of animals [16,17,18]. One reason for an improvement of growth performance caused by the MCFAs treatments is related to the increased activity of digestive enzymes and nutrient digestibility [13,19]. Furthermore, diets supplemented with MCFAs are beneficial to intestinal health and functions by improving gut barriers and microenvironment, resulting in a better host performance [10,20]. Noticeably, the excessive inclusion of MCFAs had no positive responses, even negative effects, on growth performance of suckling and weanling piglets [21], which is related to reduced feed palatability and low feed intake of animals. Therefore, there is a dose-dependent response of MCFAs supplementation on regulating growth performance of the host.

The integrity of intestinal morphology is influenced by strategies of dietary nutrients, since intestinal epithelial cells are always in a process of renewal and repair, and this normal physiology requires much energy from dietary nutrients to be kept [22]. In the current study, sodium decanoate increased villus height and a ratio of villus height to crypt depth in the ileum, but did not affect villus height and crypt depth in the colon of mice. MCFAs have a positive regulation on intestinal morphology and barrier functions of weanling piglets, as well as mice. Dietary supplementation of MCFAs increased villus height and a ratio of villus height to crypt depth but decreased crypt depth of weanling piglets [23]. MCFAs added in the diet of weanling piglets can effectively release situations of jejunal callus atrophy, epithelium cell shedding, and reduced villus height induced by lipopolysaccharides, resulting in decreasing mucosal damage of piglets and protecting immunological barrier integrity of intestinal mucosa [24,25].

Treatments of caprylic acid and nonanoic acid had no effects on the protein expression of occluding and ZO-1 in the porcine jejunal epithelial cell line IPEC-J2 challenged with *Escherichia coli* ATCC 43889 (O157:H7) [6]. On the contrary, our findings showed that a diet supplemented with sodium decanoate improved the protein expression of claudin-1 and claudin-3 in the ileum, and occludin and claudin-3 in the colon of mice. A difference in the protein expression of tight junction proteins mentioned above should be associated with sources of MCFAs and technical methods of in vitro and in vivo. A previous study reported that supplementation of MCFAs can upregulate the expression of intestinal claudin-1 protein, and downregulate the mRNA expression of activin receptor-interacting protein and mixed spectrum kinase domain-like protein, which suggests that an improvement of intestinal integrity induced by MCFAs is related to regulate RIP-3 and MLKL signaling pathways [26].

Many studies reported that plant oils rich in medium and long chain fatty acids increased the antioxidant capacity of animals, resulting in an improvement of gut health [27]. However, there is limited research on the roles of extracted MCFAs in regulating antioxidant functions of the host. Organic acids combined with MCFAs in the diet improved T-AOC but decreased MDA in the serum of weanling piglets compared with a diet containing organic acids [25]. In our study, a diet supplemented with sodium decanoate increased the concentrations of SOD and GSH-Px, but reduced the MDA content in the serum of mice. It indicates that sodium decanoate could improve antioxidant capacity and suggests that sodium decanoate can be supplemented in the diet to release the oxidant stress of animals derived from the environment, such as weaning stress and high temperature stress. Potential mechanisms of MCFAs to improve antioxidant capacity have been clarified that they act as a ligand of peroxisome proliferator-activated receptor γ (PPARγ) to activate the PPARγ signaling pathway, which is involved in adipogenic differentiation and immunological functions of the host [28,29].

MCFAs can reduce the inflammatory response by reducing levels of IL-6 and TNF-α in the serum [20]. Oral administration of MCFAs can stimulate secretion of intestinal IgA in rats injected with LPS, resulting in reduced expression of proinflammatory cytokines and chemokines [30]. However, there were no significant responses of sodium decanoate on inflammatory cytokines in the blood of mice in the current study, which is associated with sources and treatment levels of MCFAs. Furthermore, many studies have shown that MCFAs induced expression of host defense peptides in the intestine to strengthen host immunological functions [6,31]. Butyrate is a well-known HDAC inhibitor and has been shown to induce HDP gene expression primarily by acting as an HDAC inhibitor [32]. MCFAs, such as caprylic acid, can also act as an HDAC inhibitor in primary hippocampal neuron cells [33]. Many previous studies reported that mechanisms of sodium decanoate supplementation on an improvement of host immunological functions is to inhibit receptors of Toll-like receptor 4 (TLR4) and nucleotide oligomeric domain (NOD) signaling pathway, and reduce mRNA expression of tumor necrosis factor receptor, nuclear factor NF-kB, and receptor interactions protein kinase 2 (RIPK2) [26]. In addition, under high temperature stress, diets supplemented with MCFAs increased mRNA expression of heat shock protein 70 (HSP 70) in blood and liver of weanling piglets, and alleviated adverse reactions caused by heat stress [34].

MCFAs are a kind of anionic surfactant, which can destroy the bacterial membrane and enter bacterial cells to inhibit the activity of intracellular lipase [35]. On the other hand, MCFAs can reduce the pH value in the gastrointestinal tract and regulate the gut microenvironment, resulting in suppression of gut harmful microbiota [36,37]. Therefore, MCFAs have a strong antimicrobial activity and play an important role in modulating the gut microbial composition of the host. In agreement with our findings, the treatment of sodium decanoate increased the richness of microbiota community in the ileum and colon of mice. In addition, MCFAs supplemented into the diet of weaned piglets can reduce numbers of Escherichia coli and Enterococcus in the ileum and cecum, and decrease abundances of harmful bacteria such as *Escherichia coli*, *Salmonella*, and *Enterococcus* but increase a population of *Lactobacillus* in feces [38,39]. Conversely, we found that supplementation of sodium decanoate reduced an abundance of *Lactobacillus* in the ileal of mice, which may be caused by antimicrobial activity of sodium decanoate to some content. MCFAs formulated in the diets of poultry inhibited the growth of *Salmonella*, Firmicutes, and *Enterococcus* in the intestine [40,41], which agreed with our results that a diet supplemented with sodium decanoate decreased the population of Firmicutes in the ileum of mice. In mice, MCFAs supplementation could suppress proliferation of *Clostridium difficile* in the upper intestine [42]. In the present study, sodium decanoate supplemented into a diet decreased abundances of *Streptococcus*, which are a primary pathogen in humans to cause clinical symptoms of inflammation. In the present study, a diet formulated with sodium decanoate increased abundances of *Faecalibaculum* and *Bifidobacterium* in the colon of mice. *Faecalibaculum* is mainly a gut bacteria to produce VFA by fermenting non-digestable carbohydrates in diets, and *Bifidobacterium* is an acetic acid-producing bacteria in the intestine [43,44]. Therefore, the addition of sodium decanoate could increase intestinal VFA concentration by shaping gut microbial community, which is consistent with our results that sodium decanoate provided to mice increased concentrations of acetic acid, propionic acid, and butyric acid in the ileum and colon. Many studies have reported that VFA, especially butyrate, improves the functions of intestinal barrier and host immune, and is beneficial to host metabolism and physiology via activating GPRs located on the intestine and suppressing the activity of histone deacetylase [45,46]. Recently, a research also indicated that MCFAs, caprylic acid, and nonanoic acid could inhibit histone deacetylase to upregulate the expression of host defense peptides and improve the immunological barriers function of the intestine [6]. However, there is a large variation on antimicrobial activity among different sources of MCFAs. Octanoic acid and decanoic acid have stronger inhibitory responses on Gram-positive bacteria than on Gram-negative bacteria, and lauric acid mainly inhibited Gram-negative bacteria [38]. A combination of octanoic acid and decanoic acid used in the diet had a synergistic antibacterial ability (Dierick et al. 2003). Overall, sodium decanoate can increase the richness of intestinal microbial composition and abundances of *Faecalibaculum* and *Bifidobacterium* to promote VFA production, resulting in an improvement of gut health and functions in hosts.

GPRs are the largest and most diverse family of transmembrane proteins, which can be expressed in many tissues of the host, especially in intestinal tissues and epithelial cells [47]. GPR-43 is even more widely expressed among GPRs, with the highest expression in intestinal immune cells, which indicated that activation of leucocytes is mediated by GPR-43 to improve the intestinal immunological barrier and host immune functions [48,49]. Many previous studies have proved that butyrate improves the capacity of anti-inflammatory and antitumorigenic, promotes the expansion and differentiation of Tregs, and increases insulin sensitivity, energy expenditure, and secretion of PYY and GLP-1 by regulating functions of intestinal barrier mediated by activating GPR-43 [50,51]. There is no published study on the effects of MCFA on the expression of GPR-43 in the intestine. In this study, we found that the sodium decanoate treatment improved the protein expression of GPR-43 in the ileum and colon of mice, resulting in the improved intestinal barrier function and antixiodative capacity.

## 5. Conclusions

The sodium decanoate treatment improved intestinal morphology, antioxidant capacity, and intestinal barrier functions, as well as optimized gut microbial community and increased volatile fatty acid production, resulting in an improvement of gut health and growth performance of the host. Potential mechanisms of sodium decanoate on improving gut health and antioxidant capacity of the host should be associated with upregulating the expression of intestinal GPR-43, indicating that sodium decanoate can be considered as an effectlive antibiotics alternative to cure the clinical symptoms of gastrointatinal tract at weaning.

## Figures and Tables

**Table 1:** Effects of dietary sodium decanoate on growth performance of C57/BL6 mice (Exp. 2) ^1,2^

Items	CON	DA	BA	*p* Value
Initial weight, (g)	13.52 ± 0.51	13.68 ± 0.60	13.67 ± 0.55	>0.05
Final weight, (g)	27.54 ± 0.65 ^a^	28.83 ± 0.59 ^b^	28.45 ± 0.56 ^b^	<0.05
ADG, (g/day)	0.50 ± 0.01 ^a^	0.54 ± 0.01 ^b^	0.53 ± 0.01 ^b^	<0.05
ADFI, (g/day)	2.65 ± 0.15	2.62 ± 0.08	2.58 ± 0.10	>0.05
F/G	5.30 ± 0.09 ^a^	4.85 ± 0.12 ^b^	4.87 ± 0.08 ^b^	<0.05

^1^ Values are means, n = 6 replicates. SEM: Standard error of the mean. ^a,b,c^ Different superscripts within a row mean significantly different (*p* < 0.05). ^2^ Abbreviations: ADFI: Average daily feed intake; ADG: Average daily weight gain; F/G: Feed conversion ratio: The ratio of ADFI to ADG; CON: The control diet; BA: The basal diet supplemented with 0.5% sodium butyrate; DA: The basal diet supplemented with 0.5% sodium decanoate.

**Table 2:** Effects of dietary sodium caprylate on volatile fatty acids (VFAs) concentration in intestinal contents of C57/BL6 mice (Exp. 2) ^1,2.^

Variable	CON	DA	BA	*p* Value
**Ileal digesta, mg/g**				
Acetic acid	1.12 ± 0.03 ^a^	1.40 ± 0.02 ^b^	1.62 ± 0.03 ^c^	<0.05
Propionic acid	0.50 ± 0.01 ^a^	0.58 ± 0.01 ^b^	0.62 ± 0.01 ^b^	<0.05
Butyric acid	0.26 ± 0.02 ^a^	0.36 ± 0.01 ^b^	0.38 ± 0.01 ^b^	<0.05
Total VFA	1.88 ± 0.06 ^a^	2.34 ± 0.07 ^b^	2.62±0.08 ^b^	<0.05
**Colonic digesta, mg/g**				
Acetic acid	1.90 ± 0.02 ^a^	2.25 ± 0.03 ^b^	2.36 ± 0.03 ^b^	<0.05
Propionic acid	1.15 ± 0.03 ^a^	1.53 ± 0.02 ^b^	1.65 ± 0.03 ^b^	<0.05
Butyric acid	0.64 ± 0.02 ^a^	0.82 ± 0.02 ^b^	0.80 ± 0.02 ^a,b^	<0.05
Total VFA	3.09 ± 0.06 ^a^	4.60 ± 0.08 ^b^	4.81 ± 0.09 ^b^	<0.05

^1^ Values are means ± SEMs; *n* = 6 replicates. ^a,b,c^ Different superscripts within a row mean significantly different (*p* < 0.05). ^2^ Abbreviations: CON: The control diet; BA: The basal diet supplemented with 0.5% sodium butyrate; DA: The basal diet supplemented with 0.5% sodium decanoate.

## Data Availability

The raw data are availble for readers if necessary.

## Supplementary Materials

The following are available online at https://www.mdpi.com/article/10.3390/nu13082756/s1, Table S1. Nutritional composition of basal diet (%, as-fed basis). Figure S1. Cell viability of IPEC-J2 after different incubation time with different concentrations of sodium decanoate and sodium butyrate. Figure S2. Cell viability of IPEC-J2 after different incubation time with different concentrations and treatment periods of H_2_O_2_. Figure S3. Effects of sodium decanoate and sodium butyrate on morphology of IPEC-J2 cells induced by H_2_O_2_. Figure S4. Differential microbiota in the ileal and colonic digesta of mice among 3 dietary treatments. (A) Differential bacteria among control, sodium decanoate and sodium butyrate groups; (B) Differential bacteria among control, sodium decanoate and sodium butyrate groups.
