# Supplementary material for: Sodium Decanoate Improves Intestinal Epithelial Barrier and Antioxidation via Activating G Protein-Coupled Receptor-43
Source: Nutrients. 2021 Aug 11;13(8):2756. doi: 10.3390/nu13082756 (PMC8401944; doi:10.3390/nu13082756)

Supplementary Table S1. Nutritional composition of basal diet (%, as-fed basis)

Ingredients	Content
Casein, 80 Mesh	18.96
L-Cystine	0.28
Corn Starch	29.86
Maltodextrin 10	3.32
Sucrose	33.18
Cellulose, BW200	4.74
Soybean Oil	2.37
Lard	1.90
Mineral Mix M1002	0.95
DiCalcium Phosphate	1.23
Calcium Carbonate	0.52
Potassium Citrate, 1 H_2O	1.56
Vitamin Mix V10001	0.95
Choline Bitartrate	0.19
FD&C Yellow Dye #5	0.005
Total	100.00
Nutrient levels, %	
Protein	19.2
Carbohydrate	67.3
Fat	4.3
Energy, kcal/g	3.85

Note: 1 Formulated by E. A. Ulman, Ph.D., Research Diets, Inc., 8/26/98 and 3/11/99.

2 Typical analysis of cholesterol in lard = 0.95 mg/g.

Cholesterol (mg) /4057 kcal = 19

Cholesterol (mg) /kg = 18

Supplementary Figure S1. Cell viability of IPEC-J2 after different incubation time with different concentrations of sodium decanoate and sodium butyrate. Generally, 0.1, 0.5, 1, 2, and 5 mmol/L of sodium decanoate and sodium butyrate were used to select an optimal treatment concentration according to cell viability of IPEC-J2, and finally 1 mmol/L of sodium decanoate and 0.5 mmol/L of sodium butyrate were implemented to treatment normal IPEC-J2 for 24 h to determine expression of tight junction proteins. $^{*}P < 0.05$, $^{**}P < 0.01$, and $^{***}P < 0.001$, compared with untreated control cells.

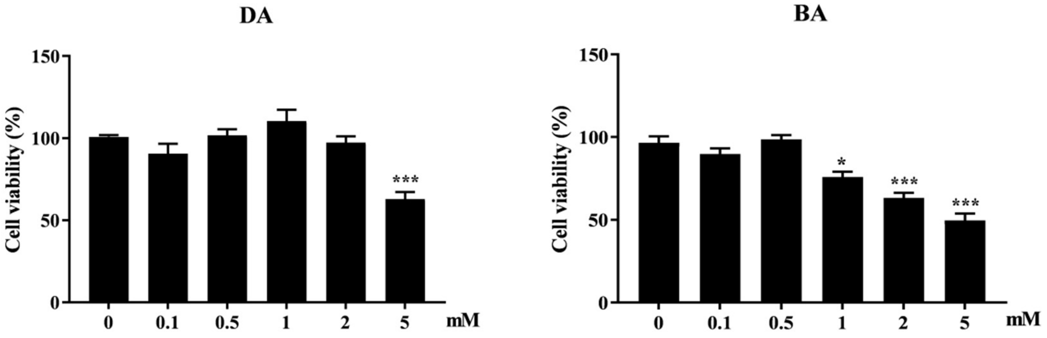

Supplementary Figure S2. Cell viability of IPEC-J2 after different incubation time with different concentrations and treatment periods of H_2O_2. IPEC-J2 cells were treated by different concentrations of 0.1, 0.3, 0.5, 0.7, 0.9, 1.1 and 1.3 μmol/L H_2O_2 and different treatment periods of 1, 2 and 4 h were used to select an optimal treatment concentration and time points. At last, IPEC-J2 cells were treated with 0.7 μmol/L H_2O_2 for 2 h to establish the oxidative damage model of the cells. $^{*}P < 0.05$, $^{**}P < 0.01$, and $^{***}P < 0.001$, compared with untreated control cells.

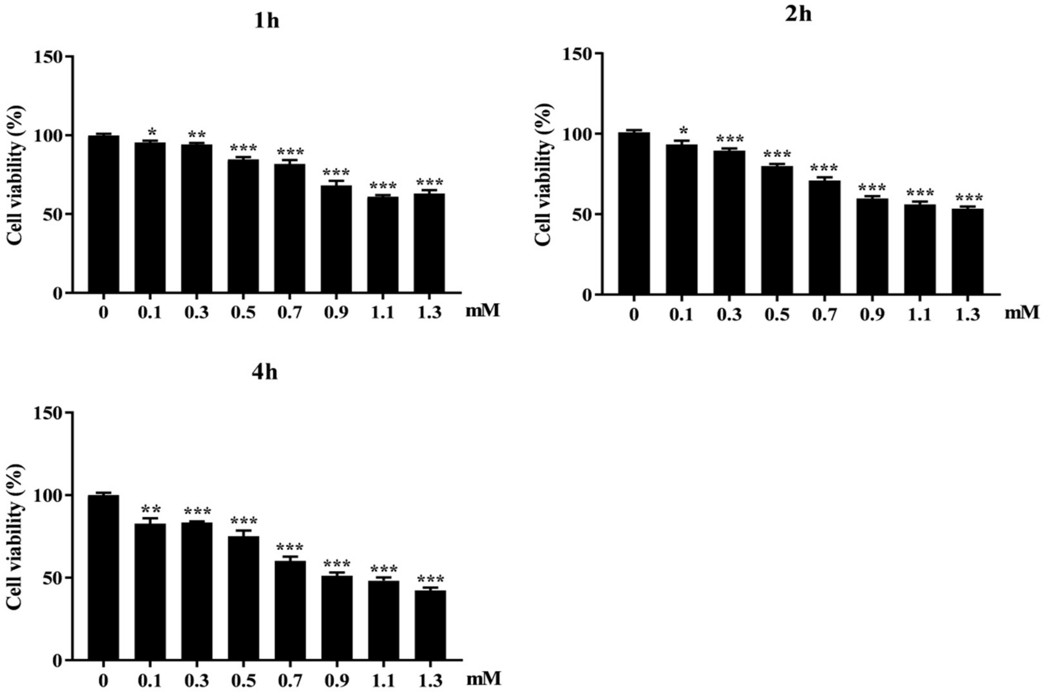

Supplementary Figure S3. Effects of sodium decanoate and sodium butyrate on morphology of IPEC-J2 cells induced by H_2O_2. A treatment concentration of 0.7 μmol/L H_2O_2 and a time treatment of 2 h were chosen to treat IPEC-J2. After H_2O_2 treatment on IPEC-J2, 1 mmol/L sodium decanoate or 0.5 mmol/L sodium butyrate was provided to culture damaged cells, in order to study the cell morphology

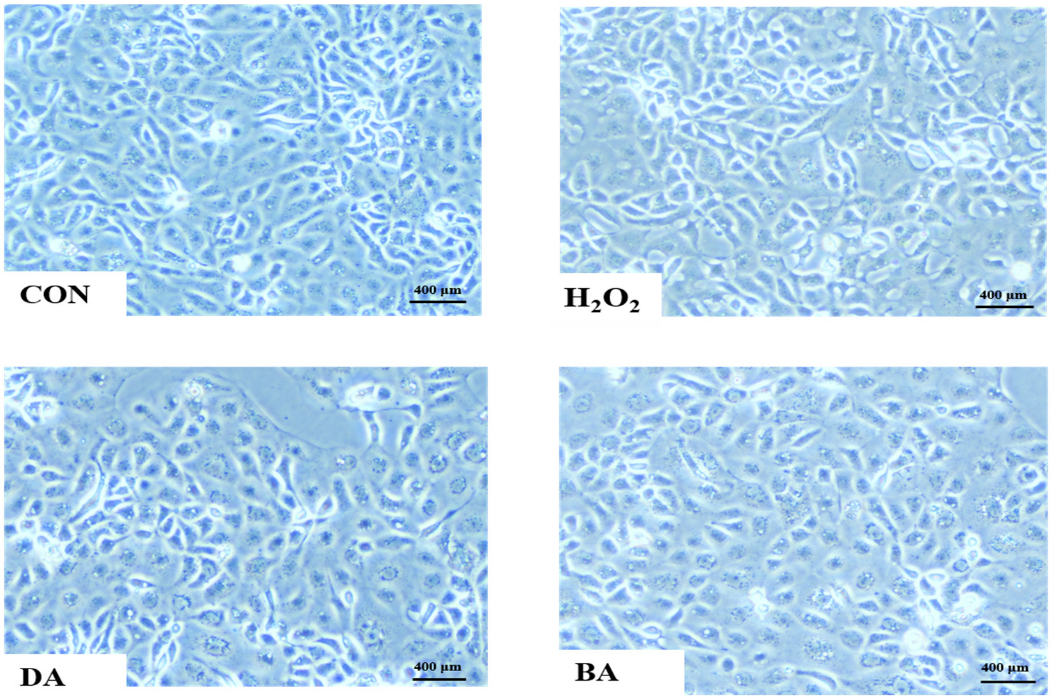

Supplementary Figure S4. Differential microbiota in the ileal and colonic digesta of mice among 3 dietary treatments. (A) Differential bacteria among control, sodium decanoate and sodium butyrate groups; (B) Differential bacteria among control, sodium decanoate and sodium butyrate groups.

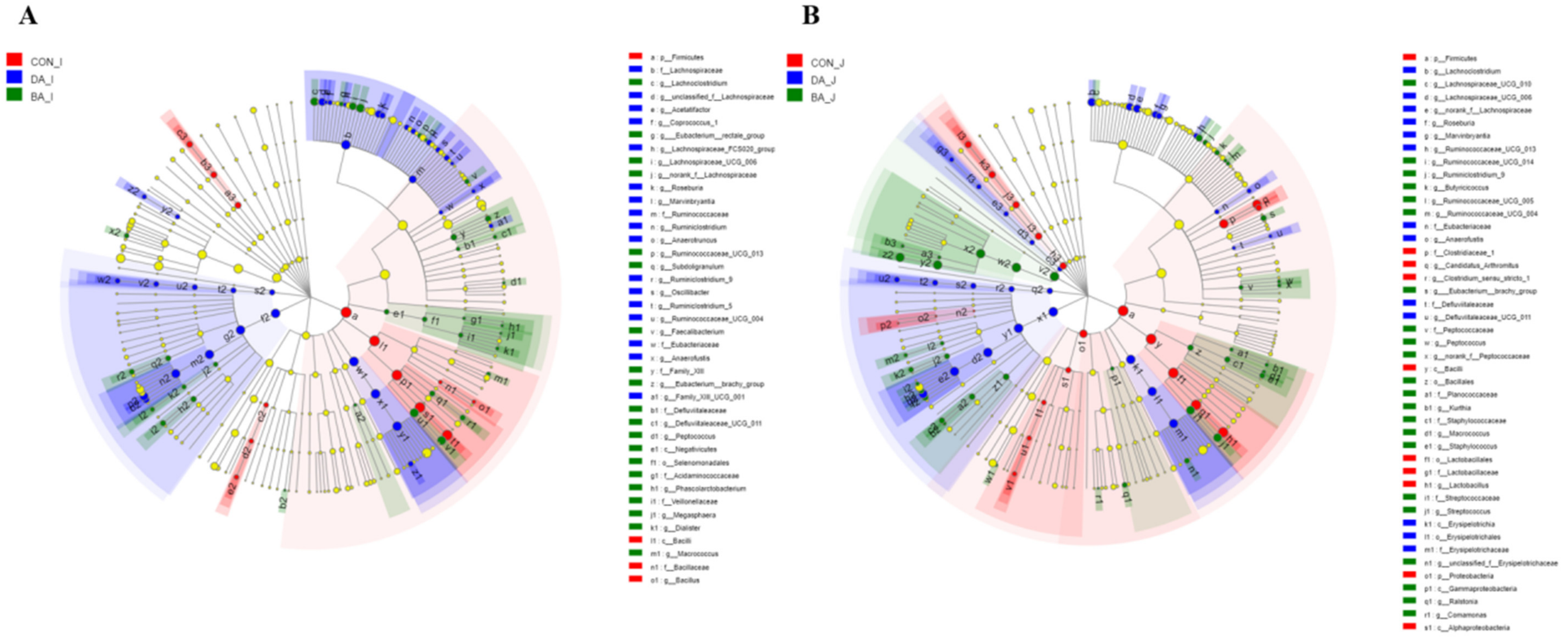

Supplement: Supplementary file 1 [file nutrients-13-02756-s001.zip › nutrients-1336050-supplementary.pdf]
